# LONG-TERM EFFECTS OF LOCAL AREA NEW DEAL WORK RELIEF IN CHILDHOOD ON LATE LIFE DEPRESSION

**DOI:** 10.1093/geroni/igae098.4235

**Published:** 2024-12-31

**Authors:** Sepideh Modrek, David Rehkopf

**Affiliations:** San Francisco State Univeresity, San Francisco, California, United States; Stanford University, Palo Alto, California, United States

## Abstract

Objective To investigate whether childhood exposure to local area New Deal emergency employment work relief activity was associated with lower depressive symptoms in late life. Methods This study utilized individual-level data from the Wisconsin Longitudinal Study (WLS) linked to the full count 1940 census. New Deal emergency employment programs were the largest non-wartime expansion in government led infrastructure, services, and employment policy in U.S. history. We used within county variation in WLS participants’ exposure to emergency employment work relief activity during childhood (ages 0-3) to examine its association with depressive symptoms in late life. We examined depressive symptoms at three ages, 53-55, 65-67, and 72-74 but with a focus on depressive symptoms at ages 72-74 as a marker for late-life depression. Results Children who lived in neighborhoods with moderate or high levels of emergency employment work relief activity exhibited 14-18% lower depressive symptom scores at ages 72-74 compared to those from neighborhoods with low activity. These findings were consistent across various measures of late-life depressive symptoms, different model specifications, and after accounting for prior depressive symptoms. Discussion The study highlights the long-term mental health benefits of economic policies aimed at mitigating concentrated economic downturns among the most impacted individuals. Childhood exposure to New Deal work relief reduced depressive symptoms in older age, particularly new onsets of depressive symptoms at ages 72-74. These results suggest social policies aimed at maintaining economic activity in downturns can have long-term positive impacts on population mental health.

